# Factors Related to Coronary Heart Disease Risk Among Men: Validation of the Framingham Risk Score

**DOI:** 10.5888/pcd11.140045

**Published:** 2014-08-14

**Authors:** Jennifer Gander, Xuemei Sui, Linda J Hazlett, Bo Cai, James R. Hébert, Steven N. Blair

**Affiliations:** Author Affiliations: Jennifer Gander, Linda J Hazlett, Bo Cai, James R. Hébert, Steven N. Blair, University of South Carolina, Columbia, South Carolina.

## Abstract

**Introduction:**

Coronary heart disease (CHD) remains a leading cause of death in the United States. The Framingham Risk Score (FRS) was developed to help clinicians in determining their patients’ CHD risk. We hypothesize that the FRS will be significantly predictive of CHD events among men in the Aerobics Center Longitudinal Study (ACLS) population.

**Methods:**

Our study consisted of 34,557 men who attended the Cooper Clinic in Dallas, Texas, for a baseline clinical examination from 1972 through 2002. CHD events included self-reported myocardial infarction or revascularization or death due to CHD. During the 12-year follow-up 587 CHD events occurred. Multivariable-adjusted hazard ratios generated from ACLS analysis were compared with the application of FRS to the Framingham Heart Study (FHS).

**Results:**

The ACLS cohort produced similar hazard ratios to the FHS. The adjusted Cox proportional hazard model revealed that men with total cholesterol of 280 mg/dL or greater were 2.21 (95% confidence interval (CI), 1.59–3.09) times more likely to have a CHD event than men with total cholesterol from 160 through 199mg/dL; men with diabetes were 1.63 (95% CI, 1.35–1.98) times more likely to experience a CHD event than men without diabetes.

**Conclusion:**

The FRS significantly predicts CHD events in the ACLS cohort. To the best of our knowledge, this is the first report of a large, single-center cohort study to validate the FRS by using extensive laboratory and clinical measurements.

## Introduction

Coronary heart disease (CHD) remains one of the leading causes of death in the United States, accounting for approximately 17% of overall national health care expenditures (1). CHD is the accrual of plaque in the arteries of the heart (2) that supply the blood for maintaining normal cardiac function. The accumulation of plaque narrows the heart’s arteries and reduces blood flow to the heart muscle. The lack of oxygen-rich blood to portions of the heart muscle leads to ischemia of myocardial tissues and consequent alteration of heart function. CHD also can be caused by the deposition of fat beneath the endothelium, reducing the elasticity of arteries (2). This arterial damage is caused by an array of significant risk factors such as hypertension (3), hypercholesterolemia (4), diabetes (5), and smoking (6). However, these risk factors are modifiable through individual and population-level behavior change; through close monitoring of cholesterol, blood glucose, and other risk factors; and by treating any of these risk factors that are above acceptable ranges with medication such as statins or insulin. As a result, many countries have experienced a decrease of CHD incidence in the past 30 years (7).

Several risk scores have been developed to provide guidance to clinicians on their patients’ risk for CHD (8,9). The Framingham Risk Score (FRS) (9,10) is the CHD risk score most widely used by clinicians across the globe (11). The FRS originated from the Framingham Heart Study (FHS), a relatively homogeneous cohort residing in Framingham, Massachusetts (9), and has been applied and validated in a variety of different populations (12,13). However, the study of Kagan et al (13) lacked complete congruency with FRS methodology, and other studies such as those of Lee et al (12) and Fried et al (14) had relatively small sample sizes. A recent publication updated the 1998 FRS and developed a new risk score that predicted an individual’s cardiovascular disease risk instead of the CHD outcome (15). For this study, we chose to investigate CHD outcomes as they comprise the majority of cardiovascular disease events (16).

Our research aims to expand on recent validation studies (17) that used the Aerobics Center Longitudinal Study (ACLS) cohort and the measured outcome of 10-year risk for CHD. ACLS provides a larger cohort to validate FRS than FHS or other previous studies, and FRS has yet to be applied to this cohort. Extensive measures of FRS components and CHD outcomes are available on the more than 40,000 participants (18) in the ACLS cohort. Our objective was to test the hypothesis that the FRS will be significantly predictive of CHD events among men in the ACLS population.

## Methods

### Study population

ACLS is an observational longitudinal study whose members were patients of the Cooper Clinic, Dallas, Texas, where they received a preventive medical examination and counseling on health behaviors during periodic visits. The Cooper Clinic serves anyone who elects to come for an examination, and patients come from all 50 states. During the patients’ medical examination, they were informed of the ACLS, asked to participate, and if they agreed to participate, they consented to follow-up surveillance. The ACLS protocol was annually reviewed and approved by the Cooper Institute’s institutional review board.

Participants were examined at least once from 1972 through 2002 at the Cooper Clinic. The cohort consisted mostly of patients in the middle and high socioeconomic groups: approximately 80% had college degrees (19). The mean baseline age of the cohort was 42 years (20) and consisted mostly of men (75%) and non-Hispanic whites (>95%).

Although ACLS is not a representative sample of the entire US population, a comparison of median values of specific physiological variables show similarity to representative population data (21).

**Figure 1:**
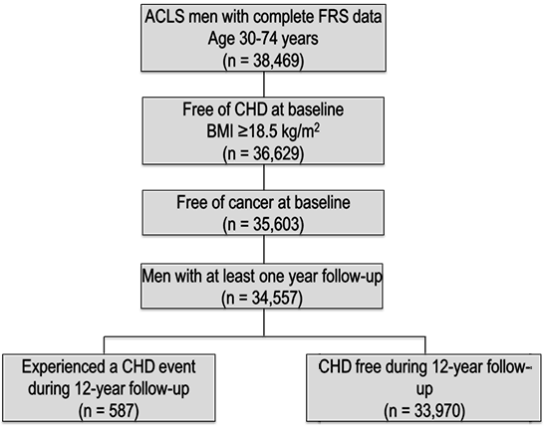
Study flow and Aerobic Center Longitudinal Study inclusion criteria depicting final sample size and coronary heart disease event frequency. Men with complete Framingham Risk Score data and body mass index ≥ 18.5 kg/m^2^ were included in the analysis. Abbreviations: FRS, Framingham Heart Study; CHD, coronary heart disease; BMI, body mass index. **Table:** 

A large number of women were enrolled in ACLS (n = 11,276); however, women were excluded from this analysis because of the small number of CHD events (n = 45) during the follow-up period. The following inclusion criteria were applied to the ACLS cohort participants for the current study: 1) age at baseline examination from 30 to 74 years, 2) complete data for outcome and predictor variables, and 3) free of CHD diagnosis or cancer diagnosis at baseline. To control for any unmeasured confounders that may have caused early drop-out, men with less than 1 year of follow-up were excluded from the study’s cohort (Figure 1).

Exclusion and Inclusion Criteria for Aerobics Center Longitudinal Study (ACLS).		Population Size After Each Criterion is Applied
1	ACLS men with complete FRS data aged 30 to 74 years	38,469
2	Free of CHD at baseline, BMI ≥ 18.5 kg/m^2^	36,629
3	Free of cancer at baseline	35,603
4	Men with at least one year of follow-up	34,557
5a	Free of coronary heart disease during the 12-year follow-up	33,970
5b	Experienced a coronary heart disease event during the 12-year follow-up period	587

Abbreviations: FRS, Framingham Heart Study; CHD, coronary heart disease; BMI, body mass index.

### Clinical examination

Trained technicians followed standardized protocols in conducting each measurement. The baseline clinical examination included a personal and family medical history, anthropometric measurements, a 12-hour fasting blood chemistry including glucose and cholesterol measurements, electrocardiogram, blood pressure assessment, and a maximal exercise test (21,22).

CHD was the primary end point being investigated. CHD was defined as the self-report of myocardial infarction or revascularization (including, bypass, coronary balloon, angioplasty, or stent) or death due to CHD. Participants reported their history of infarction or revascularization and incident date through a mail-back questionnaire administered in 1982, 1986, 1990, 1995, 1999, and 2004. Deaths among study participants were identified from the National Center for Health Statistic’s National Death Index. International Classification of Disease (ICD), Ninth Revision, codes 410.0–414.0 and Tenth Revision, codes I20–I25, were used to identify CHD as the primary cause of death. According to the FRS follow-up time definition, the maximal follow-up time was 12 years. The 12-year follow-up was used in the regression and survival analysis and then adapted to provide 10-year CHD incidence estimates.

The covariates considered for analyses in the ACLS population mimicked the variables included in the recently updated FRS (10). Hypertension was divided into 4 categories according to systolic blood pressure and diastolic blood pressure. Systolic blood pressure was categorized into 4 levels: <130 mm Hg, 130–139 mm Hg, 140–159 mm Hg, or ≥160 mm Hg, and diastolic blood pressure was categorized into 4 levels: <85 mm Hg, 85–89 mm Hg, 90–99 mm Hg, and ≥ 100 mm Hg. When an participant’s blood pressure fell into different categories for systolic and diastolic blood pressure, the higher category was chosen for categorization. For example, if a participant’s blood pressure was 130/80 (systolic blood pressure/diasystolic blood pressusre), the corresponding categories for systolic blood pressure would be 2, and the diastolic blood pressure category would be 1. To determine the hypertension category, the higher classification would be chosen and the hypertension categorization would be 2 in this example. Hypertension definition was made without regard to a participant’s use of antihypertensive medications. The definition of hypertension parallels the FRS definition (10).

Total cholesterol was grouped into four levels: <200 mg/dL, 200–239 mg/dL, 240–279 mg/dL, and ≥ 280 mg/dL. High-density lipoprotein was categorized as: <35 mg/dL, 35–59 mg/dL, and ≥ 60 mg/dL. A 12-hour fasting glucose >140 mg/dL classified an individual as having diabetes. Smoking status was dichotomized as current smoker or nonsmoker. All categorizations and definitions were analogous to FRS covariate groupings (10).

### Statistical analysis

Descriptive statistics were generated to compare the ACLS population with the FRS population. Men in each cohort were compared on mean age; percentage within each category in hypertension, total cholesterol, and HDL; percentage with diabetes, and percentage of current smokers. Univariate Cox Proportional Hazard models were performed for the CHD events and each covariate to determine each characteristic’s predictive power. Cox Survival analyses were conducted to determine the 10-year CHD risk for the ACLS male population. The fully adjusted Cox Proportional Hazard model included age, blood pressure, total cholesterol, high density lipoprotein cholesterol, diabetes diagnosis, and smoking status.

Predictive accuracy was determined through the concordance statistic (C statistic) associated with the receiver operating characteristic (ROC) curve. The ROC curve measures the discrimination power of these diagnostic markers for the CHD outcome. The Hosmer-Lemeshow statistic is used to assess calibration and is a χ2 test calculated by sorting the sample by estimated probability of success (23). The higher the C statistic, the better the prediction. A limitation of the Hosmer-Lemeshow test is that it is not recommended for sample sizes larger than 25,000. A sensitivity analysis was performed following the recommendations of Paul et al (23), and the ACLS sample (n = 34,557) and a smaller 10,000 sample cohort were randomly selected. To satisfy this limitation, the Hosmer-Lemeshow test was performed on a randomly selected cohort (n = 10,000), and *P* value of *P* > .05 represents no significant difference between predicted and observed events. All analyses were performed using SAS version 9.3.

## Results

During the 12-year follow-up period (284,572 person-years of exposure), 587 men had a CHD event. The incidence rate was 20 per 10,000 person-years. The ACLS cohort had approximately 32,000 more participants (Table 1) than the FHS, and participants were, on average, younger (*P* < .001). FHS had a higher proportion of people with diabetes (5.0%) and smokers (40.0%) than the ACLS cohort, which had 1.5% and 17.0%, respectively (*P* < .001) (Table 1).

**Table 1:** Comparison Between Demographic Characteristics of Men Free of Coronary Vascular Disease at Baseline in the Framingham Heart Study (FHS) and the Aerobics Center Longitudinal Study (ACLS)^a^

Risk Factor	Study Comparison^b^	
	FHS^c^ (n = 2,439)	ACLS (n = 34,557)
Age range, y	30–74	30–74
Mean age, y	48.30	44.82
Blood pressure, (mm Hg)		
Optimal and normal (SBP <130, DBP <85)	44.00	59.85
High normal (SBP 130–139, DBP 85–89)	20.00	16.24
Stage I hypertension (SBP 140–159, DBP 90–99)	23.00	18.98
Stage II–IV hypertension (SBP ≥160, DBP ≥100)	13.00	4.93
Total cholesterol (mg/dL)		
<160	7.00	9.34
160-199	31.00	34.36
200-239	39.00	36.67
240-279	17.00	15.10
≥280	6.00	4.53
High-density lipoprotein cholesterol (mg/dL)		
<35	19.00	16.24
35-59	70.00	70.97
≥60	11.00	12.79
Diabetes	5.00	1.52
Current smoker	40.00	16.95

Abbreviations: SBP, systolic blood pressure; DBP, diastolic blood pressure.

a Numbers are expressed as percentages unless otherwise stated.

b Independent *t* test was used to determine statistically significant difference in age between FHS and ACLS participants; proportion test calculated the statistical difference for each level of blood pressure, total cholesterol, high-density lipoprotein cholesterol, diabetes, and current smoking between FHS and ACLS participants. All proportion tests were significant with a *P* value < .001.

c FHS, Framingham Risk Score descriptive statistics referenced from D'Agostina et al (17).

When the ACLS cohort is stratified by CHD status, men who experienced a CHD event during the 12-year follow-up were significantly different on all predictor variables; that is, they were older, had higher blood pressure, and were in the top 2 categories for high-density lipoprotein cholesterol. Among those men who experienced CHD during follow-up, 4.6% had diabetes and 23.3% were smokers compared with 1.47% (*P* < .001) with diabetes and 16.8% current smokers (*P* < .001) who did not experience CHD (Table 2).

**Table 2:** Comparison Between Demographic Characteristics of Men With and Without a Coronary Heart Disease (CHD) Event in the Aerobic Center Longitudinal Study (ACLS)^a^

RISK FACTOR	CHD Event Comparison within ACLS^b^	
	No CHD (n = 33,970)	With CHD (n = 587)
Median follow-up time (IQR)	10.94 (3.82, 12.00)	5.66 (2.94, 8.93)
Age, range (years)	30-74	30-73
Mean age, y	44.70	51.91
Blood pressure, (mm Hg)		
Optimal and normal (SBP <130, DBP <85)	60.06	47.53
High normal (SBP 130–139, DBP 85–89)	16.18	19.76
Stage I hypertension (SBP 140–159, DBP 90–99)	18.85	26.41
Stage II-IV hypertension (SBP ≥160, DBP ≥100)	4.90	6.30
Total cholesterol (mg/dL)		
<160	9.44	3.92
160–199	34.62	19.59
200–239	36.60	40.37
240–279	14.88	27.60
≥280	4.46	8.52
High-density lipoprotein cholesterol (mg/dL)		
<35	16.08	25.55
35–59	71.05	66.44
≥60	12.88	8.01
Diabetes	1.47	4.60
Current smoker	16.84	23.34

Abbreviations: IQR = interquartile range; SBP, systolic blood pressure; DBP, diastolic blood pressure.

a The numbers are percentages unless otherwise stated.

b χ2 test was performed to calculate statistical difference between the group with and without CHD. All comparisons were significant at *P* < 0.05.

The covariates based on the FRS were all significant when applied to the men in ACLS (Table 3). The hazard ratios (HRs) reported from FHS by D’Agostino et al (2001) (17) were similar to the ACLS fully adjusted HRs. The fully adjusted HRs show that men with Stage I hypertension (HR = 1.41; 95% confidence interval [CI], 1.16–1.72) have significantly higher risk of CHD than men with optimal or normal blood pressure. Men with total cholesterol at or greater than 280mg/dL were more than twice (HR, 2.21; 95% CI, 1.59–3.09) as likely to have a CHD event than men with total cholesterol between 160 and 199mg/dL. Men with diabetes were 1.82 (95% CI, 1.23–2.70) times more likely to experience a CHD event than men without diabetes. Current smokers also had a significantly higher risk (HR, 1.63; 95% CI, 1.35–1.98) for CHD than nonsmokers during the 12-year follow-up.

**Table 3:** Comparison Between Hazard Ratios for Coronary Heart Disease (CHD) Events for the Framingham Heart Study (FHS) Cohort and the Aerobics Center Longitudinal Study (ACLS) Cohort

Risk Factor	FHS^a^		ACLS 12y Follow-up			
			Unadjusted		Fully Adjusted^b^	
	HR	95% CI	HR	95% CI	HR	95% CI
Age, y	1.05	1.04–1.06	1.09	1.08–1.10	1.09	1.08–1.10
Blood pressure, mm Hg						
Optimal and normal (SBP <130, DBP <85)	1.00 [Reference]		1.00 [Reference]		1.00 [Reference]	
High normal (SBP 130–139, DBP 85–89	1.31	0.98–1.76	1.66	1.33–2.06	1.33	1.07–1.66
Stage I hypertension (SBP 140–159, DBP 90–99)	1.67	1.28–2.18	1.95	1.60–2.38	1.41	1.16–1.72
Stage II-IV hypertension (SBP ≥160, DBP ≥100)	1.84	1.37–2.06	1.94	1.37–2.73	1.23	0.87–1.74
Total cholesterol (mg/dL)						
<160	0.69	0.31–1.52	0.77	0.49–1.21	0.82	0.52–1.28
160–199	1.00 [Reference]		1.00 [Reference]		1.00 [Reference]	
200–239	1.77	1.25–2.50	1.85	1.48–2.31	1.59	1.27–1.99
240–279	2.10	1.43–3.10	2.90	2.28–3.68	2.37	1.86–3.01
≥280	2.29	1.39–3.76	2.74	1.97–3.83	2.21	1.59–3.09
High density lipoprotein cholesterol (mg/dL)						
<35	1.47	1.16–1.86	1.59	1.32–1.92	1.60	1.32–1.94
35–59	1.00 [Reference]		1.00 [Reference]		1.00[Reference]	
≥60	0.56	0.37–0.83	0.66	0.49–0.90	0.60	0.44–0.81
Diabetes	1.50	1.06–2.13	3.45	2.34–5.07	1.82	1.23–2.70
Smoking status	1.68	1.37–2.06	1.60	1.32–1.93	1.63	1.35–1.98

Abbreviations: HR, hazard ratio; CI, confidence interval; SBP, systolic blood pressure; DBP, diastolic blood pressure.

a Framingham Heart Study hazard ratios are from Wilson et al (10).

b Fully adjusted model included age, blood pressure, total cholesterol, high density lipoprotein levels, diabetes diagnosis, and smoking status.

**Figure 2:**
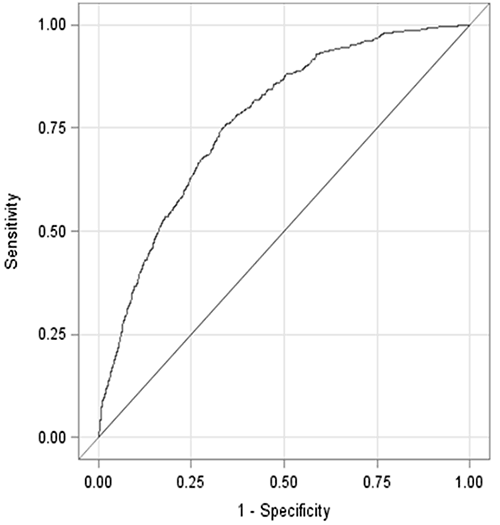
Receiver operating characteristic curve representing the predictive ability of the Framingham Risk Score applied to the Aerobics Center Longitudinal Study cohort after a 12-year follow-up. The Hosmer-Lemeshow C statistic is represented by the Area Under the Curve (C = 0.7697, 95% confidence interval, 0.7523–0.7871). **Table:** 

The C statistic (area under the curve) obtained from the receiver operating characteristic (ROC) curve was 0.7697 (95% CI, 0.7523–0.7871) (Figure 2). The Hosmer-Lemeshow test reported no significant lack of fit for the model ( *P =* .88), and we failed to reject the null hypothesis that states there is no significant difference between the predicted and observed values of the outcome variable.

Receiver Operating Characteristic Curve Association Statistics	Area Under the Curve	Standard Error	95% Confidence Interval	
**Model**	**0.7697**	**0.00889**	**0.7523**	**0.7871**

## Discussion

The FRS significantly predicts CHD events occurring during the 12-year follow-up in the ACLS, which was a much larger study than the original FHS. In addition to our main finding, we also found that age, blood pressure, total cholesterol, high-density lipoprotein cholesterol, diabetes diagnosis, and smoking status were associated with CHD events. The relative risks were congruent with the those reported from the FHS (17) and previous studies (12,13).

Elevated blood pressure creates more strain for the heart, which can cause stiffness of the heart muscle (2) or create microscopic tears in the walls that may develop into scar tissue (2). Myocardial ischemia is common among patients with hypertension (3,5), and reports from the FHS showed that hypertension was the primary cause of congestive heart failure in 35% of cases (24). Men with diabetes are also at increased risk for CHD (25), and additional research shows that people with both diabetes and hypertension have a higher incidence of heart disease than people with diabetes or hypertension alone (5).

Doyle et al published the findings of a study that examined the association between smoking and CHD (26) in two prospective studies: The FHS and the cohort from the Albany, New York, civil service study, with a combined study population of over 1,800 men without CHD (26). The Doyle et al study concluded that men with elevated systolic blood pressure and elevated total cholesterol who smoked were at a 1.8 (*P* < .05) times higher risk of premature mortality than men with elevated systolic blood pressure and elevated total cholesterol who did not smoke (26). Our findings are also in line with the Physicians’ Health Study, which reported significant effects of HDL cholesterol and total cholesterol on CHD (17).

Other researchers investigated FRS’s predictability in various populations. The Honolulu Heart Study began in 1965 with the overall goal of standardizing cardiovascular examinations (13). The cohort comprised Japanese American men born between 1900 and 1919 whose data were updated with information from their World War II Selective Service files; the final population comprised approximately 8,000 men free of CHD on baseline examination at study initiation (13). Cigarette smoking, cholesterol levels, blood pressure, sum of arm and back skinfold measurements , and uric acid levels were significant predictors of CHD; however, glucose intolerance showed no significant relationship to CHD. The lack of congruency in the significant results between the Honolulu Heart Study, FHS, and ACLS may be due to the Honolulu Heart Study population being at low risk of CHD (ie, CHD incidence observed in the Honolulu Study was about half that of the FHS).

To the best of our knowledge, this is the first large, single-center, prospective cohort to validate the FRS with the same level of precision as that of the FHS. The present study expands on previous research through the improvement of internal validity by using objectively measured clinical data.

A limitation of the ACLS cohort (similar to an FHS limitation) is the homogeneity of the study population’s sociodemographic factors. This limitation was explored through comparison analysis between ACLS and 2 large population-based cohorts; ACLS results were found to be similar to the results of the Lipid Research Clinics Prevalence Survey and the Canada Fitness Survey (27). It should be noted that ACLS homogeneity may be a strength because it improves internal validity by controlling for potential demographic confounders such as education, socioeconomic status, and race/ethnicity; however, generalizations must be made cautiously, and future research should be conducted on more diverse populations. Unlike the FHS finding, the ACLS found that having stage II–IV hypertension was not significantly associated with CHD, which may be due to the small proportion (4.93%) of the ACLS’s cohort who were in this group.

Although CHD remains one of the leading causes of death in the United States, the prevalence of CHD has decreased since 2004 (28), a reduction that can be largely attributed to better medical treatment and improvement in CHD risk profiles. The FRS was developed to assist clinicians in estimating their patients’ absolute risk for CHD (17). This study further evaluates FRS performance in the larger ACLS cohort and strictly followed the FHS methodology, which does not control for other CHD risk factors such self-rated health status (29), family history of CHD (30), and cardiorespiratory fitness (18). Future research should focus on expanding the FRS to include other modifiable risk factors. Community interventions and education programs should continue to target these CHD risk factors to further the prevention of heart disease.
